# Bortezomib inhibits Burkitt's lymphoma cell proliferation by downregulating sumoylated hnRNP K and c-Myc expression

**DOI:** 10.18632/oncotarget.4620

**Published:** 2015-07-17

**Authors:** Fat-Moon Suk, Shyr-Yi Lin, Ren-Jye Lin, Yung-Hsin Hsine, Yen-Ju Liao, Sheng-Uei Fang, Yu-Chih Liang

**Affiliations:** ^1^ Division of Gastroenterology, Department of Internal Medicine, Wan Fang Hospital, Taipei Medical University, Taipei, Taiwan; ^6^ Department of Internal Medicine, School of Medicine, College of Medicine, Taipei Medical University, Taipei, Taiwan; ^2^ Department of Primary Care Medicine, Taipei Medical University Hospital, Taipei, Taiwan; ^3^ Department of General Medicine, School of Medicine, College of Medicine, Taipei Medical University, Taipei, Taiwan; ^4^ School of Medical Laboratory Science and Biotechnology, College of Medical Science and Technology, Taipei Medical University, Taipei, Taiwan; ^5^ Division of Gastroenterology and Hepatology, Department of Internal Medicine, Taipei Medical University Hospital, Taipei, Taiwan; ^7^ Traditional Herbal Medicine Research Center, Taipei Medical University Hospital, Taipei, Taiwan

**Keywords:** Bortezomib, Burkitt's lymphoma, hnRNP K, sumoylation, c-Myc

## Abstract

Bortezomib (Velcal) was the first proteasome inhibitor to be approved by the US Food and Drug Administration to treat patients with relapsed/refractory multiple myelomas. Previous studies have demonstrated that bortezomib inhibits tumor cell proliferation and induces apoptosis by blocking the nuclear factor (NF)-κB pathway. However, the exact mechanism by which bortezomib induces cancer cell apoptosis is still not well understood. In this study, we found that bortezomib significantly inhibited cell proliferation in both human Burkitt's lymphoma CA46 and Daudi cells. Through proteomic analysis, we found that bortezomib treatment changed the expression of various proteins in distinct functional categories including unfolding protein response (UPS), RNA processing, protein targeting and biosynthesis, apoptosis, and signal transduction. Among the proteins with altered expression, hnRNP K, hnRNP H, Hsp90α, Grp78, and Hsp7C were common to both Daudi and CA46 cells. Interestingly, bortezomib treatment downregulated the expression of high-molecular-weight (HMw) hnRNP K and c-Myc but upregulated the expression of low-molecular-weight (LMw) hnRNP K. Moreover, cell proliferation was significantly correlated with high expression of HMw hnRNP K and c-Myc. HMw and LMw hnRNP K were identified as sumoylated and desumoylated hnRNP K, respectively. Using transient transfection, we found that sumoylated hnRNP K increased c-Myc expression at the translational level and contributed to cell proliferation, and that Lys422 of hnRNP K is the candidate sumoylated residue. Our results suggest that besides inhibiting the ubiquitin-proteasome pathway, bortezomib may inhibit cell proliferation by downregulating sumoylated hnRNP K and c-Myc expression in Burkitt's lymphoma cells.

## INTRODUCTION

Burkitt's lymphoma is an aggressive B-cell type of non-Hodgkin's lymphoma [1]. Burkitt's lymphoma occurs endemically in areas with malaria exposure [2] and is associated with Epstein-Barr virus (EBV) infections [3]. Most Burkitt's lymphomas overexpress the *Myc* oncogene that promotes cell cycle progression and contributes to tumor formation [4]. Overexpression of *Myc* mostly results from its translocation to an immunoglobulin gene, such as the t (8;14) (q24;q32) translocation found in approximately 85% of Burkitt's lymphomas cases. Chemotherapeutic regimens can produce good prognoses for patients with Burkitt's lymphoma [5]. High doses of methotrexate, cytosine arabinoside, and cyclophosphamide, whether or not in combination with a specific antibody (e.g., rituximab) [6], are useful chemotherapeutic drugs.

Proteasomes are multi-protein complexes and are responsible for ubiquitin-mediated protein degradation [7]. Dysregulation of proteasome activity may increase endoplasmic reticular (ER) stress, which ultimately results in apoptosis [8] and contributes to several diseases such as Parkinson's and Alzheimer's diseases [9, 10]. Inhibition of proteasome activity has been developed as a new strategy to induce tumor cell apoptosis [11]. Bortezomib, a dipeptidyl boronic acid proteasome inhibitor, was the first proteasome inhibitor to be approved by the US Food and Drug Administration (FDA) in 2003 for treating multiple myelomas and mantle cell lymphomas [12]. Besides inhibition of proteasome activity, bortezomib can also induce apoptosis by interrupting the DNA repair and unfolded protein response pathways, and by inhibiting proliferation and survival signal molecules such as PI_3_K, mitogen-activated protein kinase (MAPK), and nuclear factor (NF)-kB [13]. Bortezomib activates the c-Jun N-terminal kinase (JNK) signal pathway and increases caspase-3 and caspase-8 levels in multiple myelomas [14]. In human pancreatic cancer cells, bortezomib induces apoptosis by inhibiting the PKR-like endoplasmic reticulum kinase (PERK) and increasing ER stress [15]. Additionally, bortezomib can induce apoptosis in cancer cells by increasing the release of reactive oxygen species (ROS) and cytochrome C from mitochondria, as well as by activating caspase-3 and caspase-9 [13, 16].

Heterogeneous nuclear ribonucleoprotein K (hnRNP K) is located mainly in the nucleus [17], where it regulates DNA repair, transcription, chromatin remodeling, and telomere elongation [18]. Additionally, hnRNP K shuttles between the nucleus and the cytoplasm and participates in 3′-end modification and cleavage of pre-messenger (m)RNA and mRNA stability [19]. Previous studies have indicated that the functions and activities of hnRNP K are affected by various modifications including phosphorylation [20–22], methylation [23], ubiquitination [24], and sumoylation [25, 26]. Increased levels of hnRNP K are correlated with cancer cell proliferation and metastasis, and are implicated in the regulation of oncogene *c-Myc* transcription [27] and translation [28], as well as oncogene *c-Src* transcription [20].

Small ubiquitin-like modifiers (SUMOs) comprise a small protein family that contains at least four isoforms including SUMO-1, SUMO-2, SUMO-3, and SUMO-4. SUMOs can covalently bind to lysine residues of proteins such as p53, IκB, PML, and c-Jun, affecting their subcellular localization, transcriptional activity, and protein stability [29]. Sumoylation can be reversed by sentrin/SUMO-specific proteases (SENPs) that remove SUMO molecules from sumoylated proteins [30]. Recent reports have indicated that sumoylation regulates tumorigenesis. Ubc9, a SUMO-conjugating enzyme, is overexpressed in ovarian cancer [31], and sumoylated reptin increases metastatic potential by repressing the metastasis-suppressor gene *KAI1* [32]. Sumoylated interferon regulatory factor (IRF)-1 competes with non-sumoylated IRF-1 to inhibit cytokine-mediated apoptosis, which results in tumor cell proliferation [33].

Both hnRNP K and sumoylation have been shown to play roles in the tumorigenesis; however, whether there is a link between hnRNP K and sumoylation, and Burkitt's lymphoma, is not clear till date. In this study, we examined the influence of bortezomib on protein expression in Burkitt's lymphoma cells, and the relationship between inhibition of proliferation and downregulation of sumoylated hnRNP K by bortezomib.

## RESULTS

### Bortezomib inhibits Burkitt's lymphoma cell proliferation

**Figure 1:**
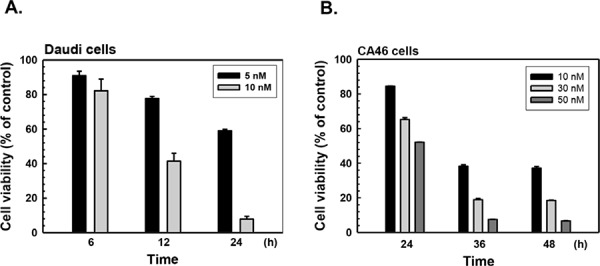
Inhibitory effects of bortezomib on the proliferation of human Burkitt's lymphoma cells **A.** Daudi and **B.** CA46 cells were treated with various concentrations of bortezomib for different periods, and the viable cells were determined by MTT assay. The data are presented as mean ± S.E. of three independent experiments.

The Daudi and CA46 cell lines are both derived from human Burkitt's lymphoma cells and express high levels of c-Myc and BCL-2. However, Daudi cells are EBV nuclear antigen (EBVA)-positive, while CA46 cells are EBVA-negative. To investigate whether bortezomib can inhibit proliferation of human Burkitt's lymphoma cells, we used both Daudi and CA46 cells. As shown in Fig. 1, bortezomib significantly decreased the viable cell numbers in dose- and time-dependent manners. The half maximal inhibitory concentration (IC_50_) of bortezomib was approximately 10 nM at 12 h in Daudi CA46 cells, and approximately 50 nM at 24 h in CA46 cells. Prolonged treatment with bortezomib at IC_50_ concentrations resulted in gradual shrinking of the cells followed by cell death. Therefore, cells were treated at IC_50_ concentrations in the proteomic analysis.

### Proteomic analysis of altered proteins in bortezomib-treated Burkitt's lymphoma cells

**Figure 2:**
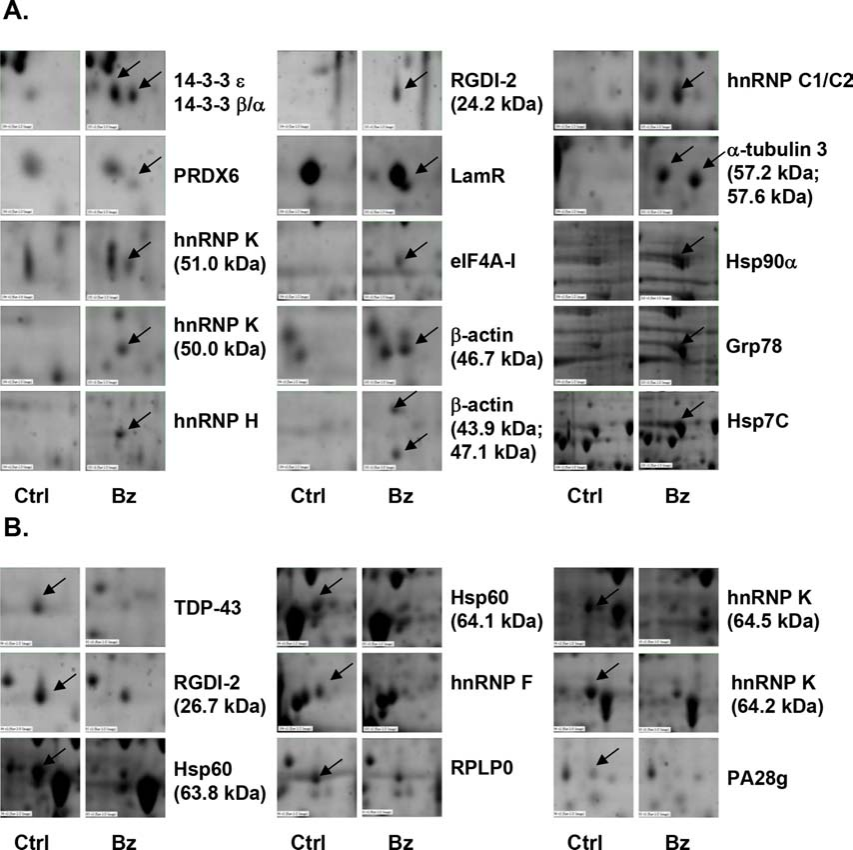
Proteomic analysis of altered proteins in bortezomib-treated human Burkitt's lymphoma cells Daudi cells were treated with 10 nM bortezomib (Bz) for 12 h, total cellular proteins were subjected to two-dimensional electrophoresis, and altered proteins were identified by MALDI-Q-TOF. **A.** Upregulated and **B.** downregulated proteins. Ctrl, control.

To investigate which proteins are altered in bortezomib-treated Burkitt's lymphoma cells, the expression profiles of bortezomib-treated CA46 and Daudi cells were analyzed by using two-dimensional electrophoresis and MALDI-TOF/MS. With a fixed threshold of 2.0, the expression of 96 proteins in Daudi cells and of 60 proteins in CA46 cells was found to significantly differ between control and bortezomib-treated cells. However, only 26 of the proteins in Daudi cells were identified by using MALDI-TOF/MS followed by a database search (Table 1). Of these 26 proteins, 17 were significantly upregulated, and 9 were significantly downregulated in bortezomib-treated cells. The altered proteins are presented in Fig. 2. In CA46 cells, 24 proteins belonging to 22 protein classes were identified, among which 19 were significantly upregulated and 5 significantly downregulated in bortezomib-treated cells (Supplementary Table S1). Five proteins were altered in both bortezomib-treated Daudi and CA46 cells, including hnRNP K, hnRNP H, Hsp90α, Grp78, and Hsp7C, which are associated with stress responses and mRNA processing. Several altered proteins were detected in multiple spots and had different molecular weights (Mws) and isoelectric points (PIs), including hnRNP K, β-actin, α-tubulin 3, RGDI-2, and Hsp60 in Daudi cells (Table 1), and Hspβ1 and hnRNP K in CA46 cells (Supplementary Table S1). In particular, different Mw forms of hnRNP K and RGDI-2 exhibited opposite changes in expression level in bortezomib-treated cells. In bortezomib-treated Daudi cells, the expression of RGDI-2 with a theoretical Mw of 23.0 kDa and a calculated Mw of 24.2 kDa was upregulated; however, RGDI-2 with a calculated Mw of 26.7 kDa was downregulated. Various Mw forms of hnRNP K (with a theoretical Mw of 51.0 kDa) were detected, which could be divided into a high-Mw (HMw) group (of around 64 kDa) and a low-Mw (LMw) group (of around 51 kDa). Interestingly, the HMw group was downregulated, while the LMw group was upregulated in bortezomib-treated Daudi and CA46 cells.

**Table 1:** List of significant altered protein identified by MALDI-Q-TOF in bortezomib-treated Daudi cells

Protein name (Calculates kDa, PI)	SWISS-PROT number	Mascot score	Change folds	Cellular location	Molecular function
14-3-3 β/α (28.5, 4.73)	P31946	56	+95.32	Cytoplasm, melanosome	Ras protein signal transduction, apoptosis
PRDX6 (23.8, 5.65)	P30041	89	+74.21	Cytoplasm, lysosome	Antioxidant activity, redox regulation
hnRNP K (51.0, 5.99; 50.0, 6.30)	P61978	39; 90	+62.77; +19.92	Cytoplasm, nucleus	hnRNP, mRNA processing
hnRNP H (57.0, 6.08)	P31943	315	+40.00	Nucleus, nucleoplasm	hnRNP, mRNA processing
RGDI-2 (24.2, 6.88)	P52566	73	+20.17	Cytoplasm	Regulates the GDP/GTP exchange
LamR (44.6, 4.70)	P08865	67	+14.74	Cytoplasm	Translational elongation, cell adhesion
eIF4A-I (49.7, 5.59)	P60842	186	+10.89	Cytoplasm	Protein biosynthesis
β-actin (47.1, 5.98; 43.9, 5.96; 46.7, 5.82)	P60709	89; 101; 249	+10.88; +5.98; +3.02	Cytoplasm, cytoskeleton	Structural protein, cell motility
hnRNP C1/C2 (41.7, 5.28)	P07910	124	+8.06	Nucleus	hnRNP, mRNA processing
α-tubulin 3 (57.2, 5.40; 57.6, 5.33)	Q71U36	228; 233	+6.33; +3.07	Cytosteleton	Microtubules
Hsp90α (96.1, 4.91)	P07900	112	+5.24	Cytoplasm, melanosome	Stress response, chaperone
Grp78 (80.6, 4.96)	P11021	237	+3.71	Endoplasmic reticulum	Stress response, anti-apoptosis
Hsp7C (74.5, 5.39)	P11142	102	+2.86	Cytoplasm, melanosome	Stress response, chaperone
14-3-3 ε (28.5, 4.67)	P62258	83	+2.29	Cytoplasm, melanosome	Adapter protein, intracellular signaling
TDP-43 (49.1, 6.05)	Q13148	160	−2.33	Nucleus	Transcription regulation, mRNA processing
RGDI-2 (26.7, 5.10)	P52566	128	−2.50	Cytoplasm	Regulates the GDP/GTP exchange
Hsp60 (63.8, 5.21; 64.1, 5.36)	P10809	90; 299	−2.95; −3.45	Mitochondrion matrix	Chaperone, regulation of apoptosis
hnRNP F (52.4, 5.41)	P52597	99	−3.21	Nucleus, nucleoplasm	hnRNP, mRNA processing
RPLP0 (39.9, 5.67)	P05388	92	−3.57	Ribonucleoprotein	Translational elongation
hnRNP K (64.5, 5.12; 64.2, 5.51)	P61978	65; 52	−3.70; −2.38	Cytoplasm, nucleus	hnRNP, mRNA processing
PA28g (33.6, 5.83)	P61289	260	−5.88	Proteasome	Immunoproteasome assembly

### Downregulation of sumoylated hnRNP K in bortezomib-treated cells

**Figure 3:**
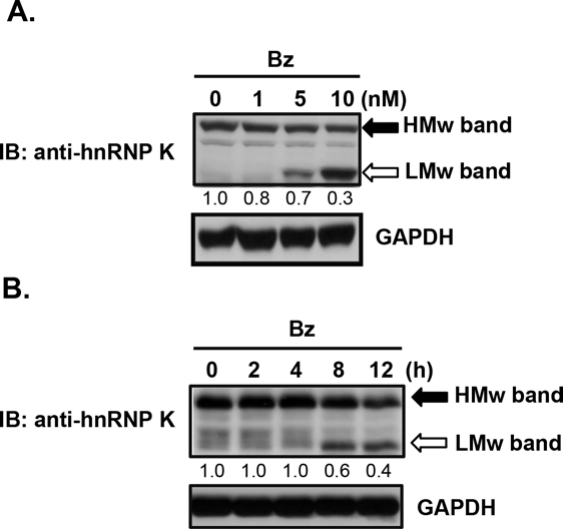
Changes in high- and low-molecular-weight hnRNP K protein expression in bortezomib-treated human Burkitt's lymphoma cells (A, B) Daudi cells were treated with **A.** various concentrations of bortezomib (Bz) for 12 h, or **B.** 10 nM bortezomib for the indicated periods. Total cellular proteins were collected to detect protein expression by western blotting. HMw, high-molecular-weight; LMw, low-molecular-weight. The numbers below the lanes indicate the relative intensities of HMw to control (defined as 1.0) proteins.

**Figure 4:**
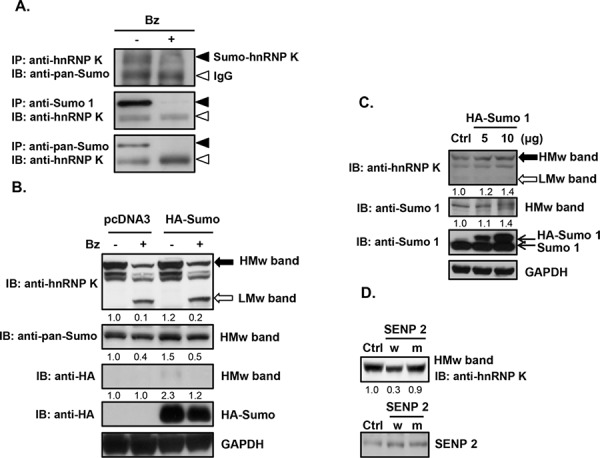
Downregulation of sumoylated hnRNP K expression in bortezomib-treated human Burkitt's lymphoma cells **A.** Daudi cells were treated with 10 nM bortezomib (Bz) for 12 h. Total cellular proteins were collected to detect protein expression by immunoprecipitation (IP) and immunoblotting (IB). Solid arrowhead, sumoylated hnRNP K (Sumo-hnRNP K); open arrowhead, heavy chain of the IgG antibody (IgG). **B.** Daudi cells were transfected with the control plasmid (pcDNA 3) or the SRa-HA-SUMO plasmid (HA-Sumo) by electroporation. After 48 h of transfection, the cells were treated with 10 nM bortezomib for 12 h, and total cellular proteins were collected to detect protein expression by western blotting. **C.** Daudi cells were transfected with 5 and 10 μg of the SRa-HA-SUMO plasmid (HA-Sumo) by electroporation. After 48 h of transfection, total proteins were collected to detect protein expression by western blotting. **D.** Daudi cells were transfected with wild-type (w) and mutant (m) plasmids of SENP2 by electroporation. After 48 h of transfection, total proteins were collected to detect protein expression by western blotting. HMw, high-molecular-weight; LMw, low-molecular-weight. The numbers below the lanes indicate the relative intensities of HMw to control (defined as 1.0) proteins.

To confirm the expression profile of hnRNP K obtained from the proteomic analysis, we treated Daudi cells with bortezomib and performed western blot analysis. As shown in Fig. 3, bortezomib treatment significantly decreased HMw but increased LMw hnRNP K protein expression in time- and dose-dependent manners. Previous studies have demonstrated that multiple post-translational modifications such as phosphorylation, methylation, ubiquitination, and sumoylation, may occur in hnRNP K [25, 26]. Most SUMO proteins are approximately 12 kDa in mass, which matched the difference between the HMw and LMw forms of hnRNP K. To ascertain hnRNP K sumoylation, we performed immunoprecipitation with an anti-hnRNP K antibody followed by immunoblotting with an anti-pan-SUMO antibody. As shown in Fig. 4A, sumoylated hnRNP K was detected in control Daudi cells, but it had decreased in bortezomib-treated cells. To confirm these findings, we performed immunoprecipitation with an anti-SUMO-1 or anti-pan-SUMO antibody followed by immunoblotting with an anti-hnRNP K antibody. The results verified that sumoylated hnRNP K was present in control Daudi cells, but decreased in bortezomib-treated cells. Next, we investigated whether the HMw band (of around 64 kDa) in Fig. 3 represented sumoylated hnRNP K. Daudi cells were transiently transfected with pcDNA 3 control plasmid or SUMO-1 expression plasmid and then treated with or without bortezomib. As shown in Fig. 4B, the HMw band was stained by the hnRNP K and pan-SUMO antibodies, and the staining was weaker in bortezomib-treated cells. Transfection of the cells with the Sra-HA-SUMO-1 plasmid increased the expression of HMw hnRNP K in a dose-dependent manner as indicated by immunoblotting with hnRNP K and SUMO-1 antibodies (Fig. 4C). Since the HMw band represented sumoylated hnRNP K, the SUMO molecules should be removed by SENP. As shown in Fig. 4D, overexpression of wild-type SENP2 significantly decreased expression of HMw hnRNP K, while overexpression of mutant SENP2 did not. These results suggested that hnRNP K sumoylation is a general event in human Burkitt's lymphoma cells, and bortezomib decreases expression of sumoylated hnRNP K.

### Sumoylated hnRNP K and c-Myc expression are associated with proliferation of Burkitt's lymphoma cells

**Figure 5:**
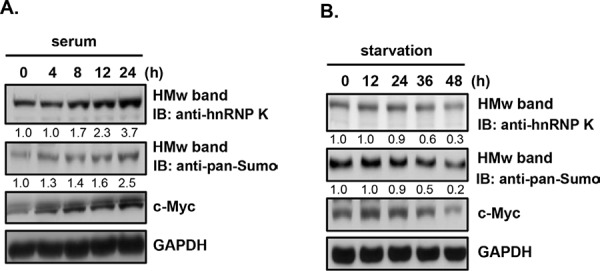
Effects of nutrition and starvation on the protein expression of sumoylated hnRNP K and c-Myc in human Burkitt's lymphoma cells **A.** Daudi cells were starved for 24 h and then treated with 10% fetal bovine serum (FBS) for the indicated periods. **B.** Daudi cells were starved for the indicated periods. Total cellular proteins were collected to detect protein expression by western blotting. HMw, high-molecular-weight; LMw, low-molecular-weight. The numbers below the lanes indicate the relative intensities of HMw to control (defined as 1.0) proteins.

Burkitt's lymphoma cells highly express the *c-Myc* oncogene [5], which is upregulated by hnRNP K both at the level of transcription and translation [27, 28]. To investigate whether expression of sumoylated hnRNP K and c-Myc is correlated with cell proliferation, we cultured Daudi cells in different nutrient conditions. Cells were either supplemented with serum to stimulate cell proliferation or deprived of serum to limit cell proliferation for different periods. As shown in Fig. 5, expression of sumoylated hnRNP K and c-Myc increased in a time-dependent manner in cells treated with serum; however, expression of sumoylated hnRNP K and c-Myc decreased under serum deprivation. These results suggested that sumoylated hnRNP K and c-Myc may be involved in regulating the proliferation of Daudi cells.

### c-Myc is upregulated by sumoylated hnRNP K at the translational level in Burkitt's lymphoma cells

**Figure 6:**
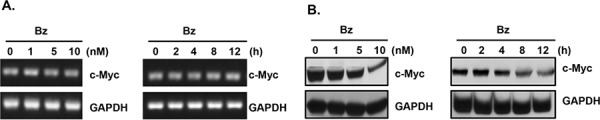
Downregulation of c-Myc protein expression in bortezomib-treated human Burkitt's lymphoma cells (A, B) Daudi cells were treated with various concentrations of bortezomib (Bz), or with 10 nM bortezomib for the indicated periods. **A.** Total RNA was extracted to detect mRNA expression by RT-PCR, and **B.** total cellular proteins were collected to detect protein expression by western blotting.

**Figure 7:**
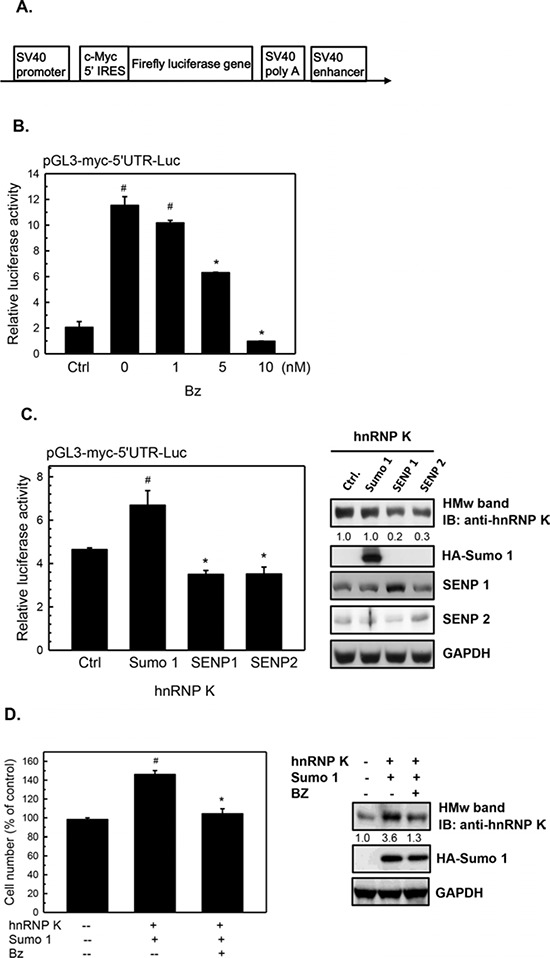
Upregulation of c-Myc protein expression by sumoylated hnRNP K in human Burkitt's lymphoma cells **A.** Schematic representation of the configuration of the pGL3-Myc-5′ UTR-Luc reporter plasmid. **B.** Daudi cells were transfected with 10 μg of the pGL3-Myc-5′ UTR-Luc reporter plasmid and the purl-TK plasmid (as an internal control) by electroporation. After 36 h of transfection, the cells were treated with various concentrations of bortezomib (Bz) for 12 h. Total cell lysates were collected to detect luciferase activities as described in “Materials and Methods”. **C.** Daudi cells were transfected with either SRa-HA-SUMO-1, Flag-SENP1, or Flag-SENP2 and hnRNP K as well as pGL3-Myc-5′ UTR-Luc and phRL-TK (as an internal control) by electroporation. After 48 h of transfection, total cell lysates were collected to detect luciferase activities as described in “Materials and Methods” and to analyze protein expression by western blotting. **D.** Daudi cells were transfected with hnRNP K and SRa-HA-SUMO-1 by electroporation. After 36 h of transfection, the cells were treated with 5 nM bortezomib (Bz) for 12 h and the viable cells were determined by MTT assay. Values are presented as the mean ± S.E. of triplicate tests. ^#^*p* < 0.05 vs. lane 1; *p < 0.05 vs. lane 2. HMw, high-molecular-weight.

To investigate whether c-Myc is regulated by sumoylated hnRNP K, we examined the mRNA and protein levels of c-Myc in bortezomib-treated cells. As shown in Fig. 6, bortezomib treatment decreased the c-Myc protein, but not the mRNA level, in dose- and time-dependent manners in Daudi cells, suggesting that c-Myc expression is regulated at the translational level. Previous studies have demonstrated that hnRNP K upregulates c-Myc expression at the translational level, where hnRNP K is bound to the internal ribosome entry sequence (IRES) region of c-Myc mRNA and increases c-Myc protein expression [28]. To investigate whether sumoylated hnRNP K upregulates c-Myc protein expression, we transiently transfected Daudi cells with the pGL3-Myc-5′ UTR-Luc reporter plasmid that contains human c-Myc IRESs and a partial c-Myc 5′ non-coding region before the firefly luciferase reporter gene (Fig. 7A). Bortezomib treatment significantly decreased the luciferase activity in a dose-dependent manner (Fig. 7B). Overexpression of SUMO-1 increased the luciferase activity; however, overexpression of SENP1 and SENP2 decreased the expression of sumoylated hnRNP K and luciferase activity (Fig. 7C). In addition, overexpression of hnRNP K and SUMO-1 significantly increased cell proliferation (~1.46-fold), while treatment with bortezomib decreased the cell proliferation induced by hnRNP K and SUMO-1 (Fig. 7D). These results suggested that c-Myc expression is upregulated by sumoylated hnRNP K at the translational level, and that sumoylated hnRNP K contributes to cell proliferation.

### Lys422 may be the major sumoylation site in hnRNP K

**Figure 8:**
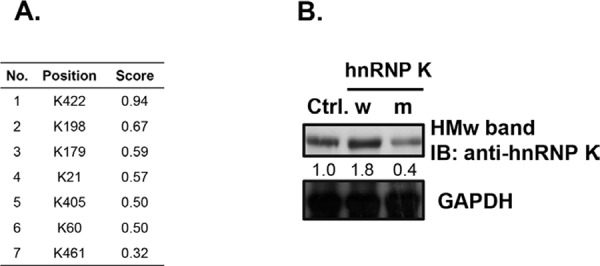
Lysine 422 is the potential sumoylation site in human hnRNP K **A.** The possible sumoylation sites of hnRNP K and the scores predicted by the SUMOplot Analysis Program. **B.** Daudi cells were transfected with the wild-type hnRNP K (w) or hnRNP K K422R mutant (m) plasmids by electroporation. After 48 h of transfection, total cell lysates were collected to detect protein expression by western blotting. HMw, high-molecular-weight. The numbers below the lanes indicate the relative intensities of HMw to control (defined as 1.0) proteins.

To predict the potential sumoylation sites in hnRNP K, we used the SUMOplot Analysis Program (http://www.abgent.com/sumoplot). Among seven potential sumoylation sites, Lys422 had the highest score (0.94) and, therefore, was considered the major sumoylation site (Fig. 8A). To verify this hypothesis, a mutant hnRNP K (Arg422) expression plasmid was constructed. As shown in Fig. 8B, overexpression of mutant hnRNP K resulted in a decrease in sumoylated hnRNP K, suggesting that Lys422 may be the major sumoylation site in hnRNP K.

## DISCUSSION

In this study, different Mw forms of hnRNP K were observed in bortezomib-treated Burkitt's lymphoma cells. The HMw and LMw forms of hnRNP K were approximately 64 kDa and 51 kDa, respectively. Further analysis indicated that the HMw form was sumoylated hnRNP K and that Lys422 might have been the sumoylation site. Previous studies have indicated that several hnRNP family proteins such as hnRNP A1, hnRNP F, and hnRNP K can be sumoylated, which may regulate mRNA metabolism [25]. Overexpression of SUMO-1 increased the protein expression of ~38-kDa hnRNP A2/B1 and ~50-kDa hnRNP A2/B1 in HepG2 cells [34]. Based on western blot results and SUMOplot predictions, a ~50-kDa form of hnRNP A2/B1 has been considered to be the sumoylated, which may have protected the protein from degradation by the ubiquitin-proteasome pathway [34]. Because the SUMO and ubiquitin modifications occurred at the same Lys residue of the target protein, they would be in competition. Similar to hnRNP A2/B1, sumoylation of hnRNP K might have protected it from degradation. In agreement with this hypothesis, normal cultured cells expressed large amounts of HMw hnRNP K, but barely expressed LMw hnRNP K. Bortezomib decreased the expression of HMw hnRNP K and increased the expression of LMw hnRNP K. These results suggested that the increase in LMw hnRNP K in response to bortezomib might be mediated by protection of hnRNP K from ubiquitin-proteasome degradation or by desumoylation of sumoylated hnRNP K. Sumoylation of hnRNP C and hnRNP M facilitated nucleocytoplasmic transport of mRNAs and decreased their binding to nucleic acids [35]. However, we demonstrated that sumoylated hnRNP K increased the translational activity of Myc 5′ UTR IRES, suggesting that sumoylation promotes binding of hnRNP K to the IRES nucleic acid segment, in contrast to hnRNP C.

Similar to ubiquitin, SUMO can attach to proteins as a single entity at one or multiple sites, to form mono- and multi-monosumoylated proteins, respectively [36]. In contrast, polysumoylation is the formation of a SUMO chain on target proteins. Monosumoylation is the major type of SUMO modification. In addition to the 64- and 51-kDa forms of hnRNP K, several other HMw bands were detected when the film was exposed for a longer time, which showed altered expression after bortezomib treatment (Supplementary Fig. S1, arrowheads). Similar findings were obtained with another proteasome inhibitor, MG132; treatment with MG132 altered the expression of different Mw forms of hnRNP K. These results suggested that endogenous hnRNP K might exist as multiple sumoylated forms, and that inhibition of proteasome activity might promote desumoylation of hnRNP K.

hnRNP K regulates protein expression at both the transcriptional and translational levels. At the transcriptional level, hnRNP K can bind to the poly(C) region of promoters of genes including *c-Src*, *eIF4E*, and *c-Myc* [37, 38]. In addition, hnRNP K can increase *FLIP* expression by binding to the poly(C) region of its promoter, leading to inhibition of tumor necrosis factor-related apoptosis-inducing ligand (TRAIL)-induced apoptosis [39]. Apart from regulating mRNA transcription rates, hnRNP K has been found to bind to various specific mRNA sequences and to regulate their translation activities. Chen et al. demonstrated that hnRNP K increased thymidine phosphorylase (TP) mRNA stability and protein translation by directly interacting with the CU-rich element of TP mRNA [40]. Another study showed binding of hnRNP K to the 3′ UTR differentiation control element (DICE) of 15-lipoxygenase (LOX) mRNA, which resulted in inhibition of LOX mRNA translation [41]. Moreover, hnRNP K upregulated c-Myc protein expression by binding to the 5′ UTR IRES of c-Myc mRNA [28]. hnRNP A1 was phosphorylated upon interleukin (IL)-6 stimulation, which increased Myc protein translation by binding to the IRES of Myc mRNA in multiple myeloma cells [42]. In this study, bortezomib decreased both sumoylated hnRNP K and c-Myc protein, but it did not change the c-Myc mRNA level. Using the pGL3-Myc-5′ UTR-Luc reporter plasmid that contains human c-Myc IRESs, we found that sumoylated hnRNP Kincreased the reporter activity, while desumoylated hnRNP K did not. These results suggested that hnRNP K regulates c-Myc expression at the translational level, and sumoylation plays an important role in binding of hnRNP Kto the IRES of c-Myc mRNA.

Recent studies have revealed that c-Myc overexpression indicates poor prognosis in multiple myelomas [43]. Our finding that bortezomib inhibits c-Myc expression might increase our understanding of the action mechanisms of bortezomib in clinical therapies of multiple myelomas and why multiple c-Myc-overexpressing myelomas respond differently to bortezomib compared to dexamethasone [44]. We found that sumoylated hnRNP K positively regulates c-Myc expression, which contributed to cell proliferation. Previous studies have indicated that combined treatment with bortezomib and other drugs such as anti-β2-microglobulin monoclonal antibody [45] and the DNA methyltransferase inhibitor decitabine [46] overcomes bortezomib drug resistance in multiple myeloma and mantle cell lymphoma, respectively. Therefore, treatment with sumoylation inhibitors alone or in combination with bortezomib may be useful in novel therapeutic strategies for multiple myelomas and Burkitt's lymphoma in future.

## MATERIALS AND METHODS

### Materials

Bortezomib was purchased from Janssen-Cilag (Buckinghamshire, UK) and was dissolved in dimethyl sulfoxide (DMSO). Anti-hnRNP K antibody was purchased from Cell Signaling Technology (New England Biolabs, Schwalbach, Germany) and Santa Cruz Biotechnology (Santa Cruz, Dallas, TX), anti-pan-SUMO, anti-SUMO-1, anti-SENP1, and anti-SENP2 antibodies were purchased from Abcam (Cambridge, MA), anti-c-Myc and anti-HA antibodies were purchased from Cell Signaling Technology, and anti-GAPDH antibody was purchased from GeneTex (Irvine, CA).

### Cell culture and viability assay

Human Burkitt's lymphoma CA46 (BCRC 60511) and Daudi (BCRC 60192) cells were obtained from the Food Industry Research and Development Institute (Hsinchu, Taiwan). Both cell lines were cultured in RPMI-1640 medium supplemented with 10% heat-inactivated fetal bovine serum (FBS, Life Technologies, Grand Island, NY), and maintained in a humidified incubator at 37°C with 5% CO_2_. CA46 and Daudi cells were cultured in 6-well plates and treated with various concentrations of bortezomib for 24 h, and the cell viability was determined by the 3-(4, 5)-dimethylthiahiazo(−z-y1)-3, 5-diphenytetrazoliumromide (MTT, Sigma Chemical, St. Louis, MO) assay as described previously [47].

### Western blotting

Total cellular proteins (30–50 μg) were fractionated by sodium dodecylsulfate-polyacrylamide gel electrophoresis (SDS-PAGE). After the proteins had been transferred onto a polyvinylidene difluoride membrane (Merck Millipore, Billerica, MA), the membrane was blocked in 1% bovine serum albumin (BSA) or 10% defatted milk for 1 h before incubation with the primary antibodies overnight at 4°C. Antigen-antibody complexes were detected using secondary antibodies conjugated to horseradish peroxidase (HRP) and visualized using enhanced chemiluminescence (ECL; GE Healthcare, Piscataway, NJ) [48].

### Reverse transcription-polymerase chain reaction (RT-PCR)

Total RNA was isolated from cultured cells and complementary (c)DNA was prepared as described previously [49]. c-Myc and glyceraldehyde-3-phosphate dehydrogenase (GAPDH) cDNAs were amplified in reactions mixtures comprising 500 ng of total cDNA in 100 mM Tris-HCl buffer (at pH 8.3) containing 500 mM KCl, 15 mM MgCl_2_, 0.1% gelatin, 200 μM of each dNTP, and 50 units/mL of SuperTaq™ DNA polymerase (Ambion, Austin, TX) and the following oligonucleotide primers: 5′-AGCCCACTGGTCCTCAAGA-3′ and 5′-CCTCTTACAGTTCTCCGCTTG-3′ for c-Myc, and 5′-TGAAGGTCGGTGTGAACGGATTTGGC-3′ and 5′-CATGTAGGCCATGAGGTCCACCAC-3′ for GAPDH. Thermal cycling conditions were as follows: 94°C for 5 min, followed by 25 cycles at 94°C for 1 min, 50°C for 1 min, and 72°C for 1 min, and a final extension at 72°C for 8 min. PCR products were separated on 1.2% agarose gels and stained with SYBR Green dye.

### Plasmids and transient transfection

Gene expression plasmids SRa-HA-SUMO-1 (no. 17359), Flag-SENP1 (no. 17357), and Flag-SENP2 (no. 18047) were obtained from Addgene (Cambridge, MA). The hnRNP K expression plasmid pCMV6-XL5-hnRNP K (sc107869) was purchased from OriGene Technologies (Rockville, MD), and the hnRNP K mutant (K422R) expression plasmid was obtained from Yeastern Biotech (New Taipei City, Taiwan). The pGL3-Myc-5′ UTR-Luc reporter plasmid containing human c-Myc IRESs, a partial c-Myc 5′ non-coding region, and the firefly luciferase gene was generously provided by Professor Anne E. Willis (University of Nottingham, Nottingham, UK).

For gene overexpression, 5 × 10^6^ cells were transiently transfected with 10 μg of plasmid by electroporation at 1000 V for 40 ms. For the reporter gene assay, cells were transiently transfected with the pGL3-Myc-5′ UTR-Luc reporter plasmid and phRL-TK (Promega, Madison, WI) as an internal control plasmid as per the above conditions and then seeded in a 24-well plate. Drug-treated cell lysates were collected, and luciferase activity was detected using the Dual-Luciferase^®^ Reporter Assay System (Promega) and a Plate Chameleon Multilabel plate reader (HIDEX OY, Turku, Finland) according to the manufacturer's instructions. Luciferase activities of the reported plasmid were normalized to luciferase activities of the internal control plasmid [50].

### Two-dimensional electrophoresis and gel imaging

Cells were lysed in solubilizing buffer (7 M urea, 4% CHAPS, and 2 M thiourea; Amersham Biosciences, Piscataway, NJ) and total cell lysates were collected according to the manufacturer's instructions. Protein samples were added to an Immobiline^TM^ DryStrip (pH 3–10, 13 cm) (Amersham Biosciences), rehydrated at 50 μA/strip and 30 V for 12 h. Isoelectric focusing (IEF) electrophoresis was carried out using the Ettan IPGphor system (Amersham Biosciences) for another 12 h. The linear voltage gradient conditions used were as follows: from 0 to 250 V for 250 Vh, 500 V for 500 Vh, 1000 V for 1000 Vh, 1500 V for 1500 Vh, 2000 V for 2000 Vh, 3000 V for 3000 Vh, 6000 V for 6000 Vh, and a final phase of 8000 V for 40, 000 Vh. Then, the Immobiline^TM^ DryStrip was placed on a 10% sodium dodecylsulfate polyacrylamide gel and second-dimension electrophoresis was performed. The gels were fixed with 10% methanol and 7% acetic acid and stained with Sypro-Ruby dye (Sigma) for 3 h.

Gel images were captured using a Typhoon 9400 densitometer and analyzed with PDQuest version 7.3.1 software (GE Healthcare). Protein spot detection and matching were performed by both automated function and manual designation. Spot intensities were obtained using the quantitative and qualitative modes and normalized to the background. Protein spot intensities with at least 2-fold difference compared to the control were considered significantly different. Protein spots of interest were collected, gel-digested by trypsin, and subjected to MALDI-TOF/MS analysis.

### Matrix-assisted laser desorption/ionization time-of-flight mass spectrometry (MALDI-TOF/MS) and analysis of peptide sequences

Selected protein spots were subjected to concerted MALDI peptide mass fingerprinting (PMF) and collision-induced dissociation (CID) MS/MS analysis for protein identification using a dedicated Q-Tof Ultima™ MALDI instrument (Micromass, Manchester, UK). The instrument system was operated under MassLynx 4.0, and raw MS data were processed for database searching using ProteinLynx Global Server 2.0. All MS and MS/MS raw data were processed with Raw2MSM and searched against a target protein sequence database using the Mascot Daemon 2.2 server. Search criteria used were as follows: trypsin digestion; variable modifications set as carbamidomethyl (Cys) and oxidation (Met); up to two missed cleavages allowed; and a mass accuracy of 10 ppm for the parent ion and 0.60 Da for fragment ions.

### Statistical analysis

All experiments were repeated at least three times. The statistical significance of differences was determined using Student's *t*-test in SPSS (IBM SPSS Statistics, Chicago, IL), with significance at *p* < 0.05

## CONCLUSIONS

We showed that several aspects of proteins can be regulated by bortezomib, in particular, it downregulated the expression of sumoylated hnRNP K and c-Myc in human Burkitt's lymphoma cells. Sumoylated hnRNP K positively regulated c-Myc expression at the translational level, and downregulation of sumoylated hnRNP K by bortezomib resulted in a decrease in c-Myc oncogene expression and subsequent inhibition of cell proliferation. The Lys422Arg mutation decreased the level of sumoylated hnRNP K, suggesting that hnRNP K can be modified by SUMO and that Lys422 might be an important sumoylation site in hnRNP K

## SUPPLEMENTARY FIGURE AND TABLE


