# Supplementary material for: Bortezomib inhibits Burkitt's lymphoma cell proliferation by downregulating sumoylated hnRNP K and c-Myc expression
Source: Oncotarget. 2015 Jul 17;6(28):25988–6001. doi: 10.18632/oncotarget.4620 (PMC4694880; doi:10.18632/oncotarget.4620)
Supplement: Supplementary file 1 [file oncotarget-06-25988-s001.pdf]

SUPPLEMENTARY FIGURE AND TABLE

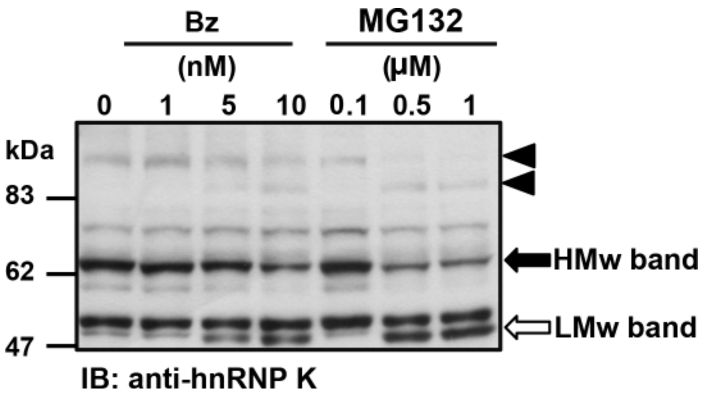

Supplementary Figure S1: Changes of several different molecular-weight hnRNP K protein expressions in proteasome inhibitors-treated human Burkitt's lymphoma cells. Daudi cells were treated with various concentrations of bortezomib (Bz) or MG-132 for 12 h. Total proteins were collected to detect protein expressions by western lotting. Arrowheads indicated the possible multiple sumoylation types of hnRNP K. HMw, high-molecular-weight; LMw, low-molecular-weight.

Supplementary Table S1. List of significant altered protein identified by MALDI-Q-TOF in bortezomib-treated CA46 cells

Protein name (Calculates kDa, PI)	SWISS-PROT number	Mascot score	Change folds	Cellular location	Molecular function
Hsp70-1/2 (79.5, 5.52)	P08107	171	+767.05	Cytoplasm, nucleus, mito	Stress response, chaperone, anti-apoptosis
Hspβ1 (29.3, 5.35; 29.3, 5.66)	P04792	86; 96	+29.20; +2.09	Cytoplasm, nucleus	Stress resistance, anti-apoptosis
Orp-150 (137.7, 5.18)	Q9Y4L1	231	+25.46	Endoplasmic reticulum	Stress response, chaperone
Ezrin (92.5, 6.60)	P15311	130	+14.70	Cytoskeleton	Cell shape
Grp78 (85.8, 4.97)	P11021	267	+7.94	Endoplasmic reticulum	Stress response, anti-apoptosis
Hsp7C (81.1, 5.40)	P11142	130	+7.24	Cytoplasm, melanosome	Stress response, chaperone
Hsp90α (100.5, 4.93)	P07900	142	+6.93	Cytoplasm, melanosome	Stress response, chaperone
hnRNP H (56.8, 6.07)	P31943	88	+4.61	Nucleus, nucleoplasm	hnRNP, mRNA processing
PSMB4 (32.6, 5.75)	P28070	72	+4.55	Cytoplasm, nucleus	Proteasome
Gβ2 (37.9, 5.55)	P62879	99	+4.42	G proteins units	Transducer, transmembrane signaling
Hsp70RY (107.9, 5.22)	P34932	136	+3.57	Cytoplasm	Stress response
hnRNP K (51.0, 5.86)	P61978	49	+3.46	Cytoplasm, nucleus	hnRNP, mRNA processing
EF-2 (111.5, 7.4)	P13639	65	+3.22	Cytoplasm	Protein biosynthesis
TerATPase (118.1, 5.14)	P55072	45	+2.68	Cytoplasm, nucleus	Protein transport
TrpRS (60.1, 6.10)	P23381	166	+2.37	Cytoplasm	Protein biosynthesis
hnRNP L (70.6, 7.62)	P14866	63	+2.35	Nucleus, nucleoplasm	hnRNP, mRNA processing
AlaRS (121.9, 5.41)	P49588	216	+2.28	Cytoplasm	Protein biosynthesis
hnRNP K (65.5, 5.23; 66.6, 5.50)	P61978	57; 53	−1.42; −1.24	Cytoplasm, nucleus	hnRNP, mRNA processing
PSET (44.1, 4.20)	Q01105	161	−2.10	Cytoplasm, ER lumen, nucleus	Apoptosis, transcription
NAC-α (36.0, 4.40)	Q13765	141	−2.20	Cytoplasm, nucleus	Protein transport
PaCAP (20.2, 5.39)	Q8WU39	48	−2.40	Cytoplasm	Apoptosis
PM20D2 (55.8, 5.76)	Q8IYS1	75	−5.20	Unknow	Unknow
